# Taxonomic diversity of sputum microbiome in lung cancer patients and its relationship with chromosomal aberrations in blood lymphocytes

**DOI:** 10.1038/s41598-020-66654-x

**Published:** 2020-06-15

**Authors:** V. G. Druzhinin, L. V. Matskova, P. S. Demenkov, E. D. Baranova, V. P. Volobaev, V. I. Minina, S. V. Apalko, M. A. Churina, S. A. Romanyuk, S. G. Shcherbak, V. I. Ivanov, A. V. Larionov

**Affiliations:** 1 Kemerovo State University, Kemerovo, Russian Federation; 2 Institute of Living Systems Immanuel Kant Baltic Federal University, Kaliningrad, Russian Federation; 3 Department of Microbiology, Tumor Biology and Cell Biology (MTC), Stockholm, Sweden; 4 Institute of Cytology and Genetics SB RAS, Novosibirsk, Russian Federation; 5 Institute of Human Ecology, Federal Research Center of Coal and Coal Chemistry of Siberian Branch of the Russian Academy of Sciences, Kemerovo, Russian Federation; 6 City Hospital #40, St. Petersburg, Russian Federation

**Keywords:** Microbiome, Non-small-cell lung cancer, Lung cancer, Non-small-cell lung cancer

## Abstract

Here we report a pilot-sized study to compare the taxonomic composition of sputum microbiome in 17 newly-diagnosed lung cancer (LC) patients and 17 controls. Another object was to compare the representation of individual bacterial genera and species in sputum with the frequency of chromosomal aberrations in the blood lymphocytes of LC patients and in controls. Both groups were male; average age 56.1 ± 11.5 in patients and 55.7 ± 4.1 in controls. Differences in the species composition of bacterial communities in LC patients and controls were significant (pseudo-F = 1.94; p = 0.005). Increased prevalence in LC patients was detected for the genera *Haemophilus* and *Bergeyella*; whereas a decrease was observed for the genera *Atopobium*, *Stomatobaculum*, *Treponema* and *Porphyromonas*. Donors with high frequencies of chromosomal aberrations had a significant reduction in the microbiome of representatives of the genus *Atopobium* in the microbiome and a simultaneous increase in representatives of the species *Alloprevotella* compared to donors with a low level of chromosomal aberrations in lymphocytes. Thus, a comparison of the bacterial composition in the sputum of donors with cytogenetic damages in theirs lymphocytes, warrants further investigations on the potential role of microorganisms in the process of mutagenesis in somatic cells of the host body.

## Introduction

Lung cancer (LC) is the most common malignant tumor and the leading cause of death in the world. In particular, deaths from LC in men account for about 1/3 of deaths for all malignant neoplasms^1^. The treatment of LC, despite the enormous efforts reflected in of basic and clinical studies, still shows unsatisfactory results on survival and prevention programs show little effect on its incidence. Complex mechanisms of pathological changes and some key biomarkers of LC are recognized but there is a significant gap in the understanding of their mechanisms of theirs action in the course of LC development and progression. Among causative factors in the etiology of LC, smoking is certainly plays a leading role^2^. At the same time, 15 to 25% of all cases of lung cancer are observed in never-smoking patients. Among women, this figure reaches 53%^3^. Therefore, in addition to smoking, there are factors that can significantly affect the risk of developing LC.

In addition to the effects of hereditary components in the causation of LC, a number of contributory factors have been reported: passive smoking^4^, air pollution^5^, professional carcinogen exposition^6^, chronic exposure to high doses of radon^7^ and, recently, the composition of the lung microbiome^8^.

In connection with the recent development of metagenomic studies, information has been rapidly accumulated regarding the presence and diversity of the microbiota populating the lungs and the entire respiratory tract.

It was noted that alpha diversity (richness and uniform distribution of taxa in samples) is significantly higher in non-cancerous lung tissues than in tumor lung tissues^9,10^, while community similarity (beta diversity) varies widely. Researchers of the LC microbiota agree that *Firmicutes* accounts for the main contribution to the development of LC^11–13^. At the level of taxa below type, the results today are mixed. In most cases, scientists have observed correlations for some individual genera. In particular, two genera: *Veillonella* and *Megasphaera* were noted as possible LC biomarkers^13^. Erb-Downward and colleagues observed a link between *Acidovorax* and small cell carcinoma^14^, other researchers attribute an increased risk of developing LC to the detection of *Granulicatella*^10,15^, *Abiotrophia*^10^, *Streptococcus*^10,12^, *Haemophilus influenzae*, *Enterobacter spp*., *Escherichia coli*^16^, *Capnocytophaga*, *Selenomonas*, *Veillonella*, *Neisseria*^17^. Given the inconsistency of the previously obtained results, and taking also into account the fact that the taxonomic composition of the respiratory microbiota depends on environmental factors, it is important to continue research, including the analysis of microbiota in patients with LC from different regions of the world. This is especially true in regions with high levels of environmental pollution, for example, due to coal burning, as was previously shown for one of the provinces of China^18^.

It was shown that changes that offset the balance in the community of microorganisms inhabiting the lungs lead to the predominance of *Haemophilus influenza, Acidovorax, Klebsiella, Moraxella catarrhalis, Mycobacterium tuberculosis*, and *Granulicatella adiacens*^19^.

It is also known that inflammatory reactions can be triggered by opportunistic species such as

*Enterobacter spp*., *E. coli*, *Pneumococcus*^16^, *Legionella*^20^ and *Moraxella*^21^. An equally important contribution to the development of LC can be made by microbiota acting on Th17-positive T-helpers, responsible for a balanced immune response and necessary for the control of autoimmune reactions. It was shown that commensal bacteria have a direct effect on the Th17-dependent pathway and calcineurin expression^22^.

Another, no less important mechanism of microbiota-induced carcinogenesis is the genotoxic properties of many bacterial metabolites. Bacterial genotoxins, such as colibactin, a cytolethal distending toxin (CDT) and others have been identified as compounds that directly damage DNA in host cells^23^. In other cases, mutagenesis in the cells of the host organism is associated with the formation of DNA-reactive metabolites due to bacterial activity, the formation of radicals, or the immune modulation of the host cells^24^. This is a mechanism of action for *Helicobacter pylori*, *Pseudomonas aeruginosa*, *Enterococcus faecalis*, *Shigella flexneri*, *Bacteroides fragilis*, *Neisseria gonorrhoeae*, *Listeria monocytogenes*, *Chlamydia trachomatis* and others. This list is clearly not exhaustive and will be updated over time.

In general, one can suggested that bacteria use different strategies to ensure their survival and replication, which includes inhibiting DNA repair of host cells, contributing to the survival of infected cells, despite the presence of DNA damage^25^. The genotoxic potential of bacterial microbiota is the basis for the hypothesis that the stability of the genome of somatic human cells, especially under the influence of genotoxic and carcinogenic factors, can directly (or indirectly) depends on the taxonomic composition of the bacterial community.

To test this hypothesis, we have undertaken efforts to analyze the taxonomic composition of the microbiota in the sputum of LC patients and healthy donors living in the environmentally challenged coal-mining region of Western Siberia (Kuzbass), Russia.

Another object was to correlate the representation of individual bacterial genera and species in sputum with the frequency of chromosomal aberrations (CA) in the blood lymphocytes of LC patients and in controls.

## Methods

### Cohort information

The composition of the bacterial microbiome was studied in 17 patients with newly-diagnosed LC (male only, average age 56.1 ± 11.5 years) who were admitted to the Kemerovo Regional Oncology Center (Kemerovo, Russian Federation) and 17 healthy male donors, residents of Kemerovo (average age 55.7 ± 4.1 years). There were no differences in mean age between patients and control (p = 0.062). Among LC patients 70.6% were active smokers, among the controls, 64.7%. A summary of the information regarding LC and control subjects is shown in Table 1. An individual questionnaire was filled out for each survey participant, containing information about the place and date of birth, profession, exposure to occupational hazards, health status, diet features, taking medications (the use of antibiotics three months before the study), X-ray procedures, and bad habits (smoking and drinking status). For LC patients, the results of clinical and histological analyses were additionally taken into account. The distribution of LC diagnoses of patients analyzed was: squamous cell carcinoma −5 (29.4%); adenocarcinoma −5 (29.4%); large cell lung carcinoma −3 (17.7%); 4 (23.5%) other forms LC: mesenchymal, non-small cell undifferentiated. In addition, for each patient, the stage of the disease was determined in accordance with the TNM classification^26^. In accordance with this, 6 patients (35.3%) had stage I-II, and 11 patients (64.7%) stage III-IV of the disease. Additionally, metastases in distant organs were present in 23.5% of LC patients.

**Table 1:** Characteristics of the study cohorts.

Variables	Lung cancer patients (case), n = 17	Healthy Kemerovo residents (control), n = 17
Age (years) (mean ± SD)	56.1 ± 11.5*	55.7 ± 4.1
**Smoking status (%):**		
Smokers	70.6	64.7
Non-smokers	29.4	35.3
Cancer type* (%):		
Squamous cell lung cancer	29.4	
Adenocarcinoma	29.4	
Large cell lung carcinoma	17.7	
Other lung cancers	23.5	
TNM** (%):		
I, II	35.3	
III, IV	64.7	

*Histological type; **tumor-node-metastasis.

### Ethics statement

All procedures undertaken were in accordance with the ethical standards of the Helsinki Declaration (1964 and amended 2008) of the World Medical Association. All participants (patients and controls) were informed about the aim, methodology and possible risks of the study; informed consent was signed by each donor. The design of this study was approved by the Ethics Committee of the Kemerovo State University.

### Sample collection, process and storage

To analyze the composition of the microbiome of the respiratory tract, sputum samples obtained from LC patients and controls were used. The sputum samples from patients were obtained prior to all diagnostic or therapeutic procedures. Sputum samples were collected non-invasively through participant-induced coughing (i.e., without induction) and represent the oropharyngeal secretion. The resulting samples were immediately placed in sterile plastic vials and frozen (−20 °C). Frozen samples were transported to the laboratory and stored at −80 °C.

### Analysis of chromosomal aberrations

Baseline frequencies of CA were studied in the same set of lung cancer patients and controls, from whom sputum microbiome was analyzed. The metaphase chromosomes spreads were prepared using the standard semi-micro-method^27^. The volumes of 1 mL of blood, 0.1 mL of phytohemagglutinin (Pan-Eco, Moscow), 9 mL of RPMI 1640 medium (Pan-Eco, Moscow), and 2 mL of fetal calf serum (HyClone) were added to the culture flask. The duration of the cultivation was 48 h.

Subsequently, colchicine (PanEco, Moscow, Russian Federation) was added to the culture at a final concentration of 0.5 μg/ml, and the flasks were placed in a 37 C incubator for 2 h. At the end of the incubation cycle, the cell cultures were treated with a hypotonic solution of 0.55% KCl for 20 min at 37 °C. The fixation of the material was performed in three changes of cooled fresh Carnoy’s fixative solution (methanol and glacial acetic acid at 3: 1 ratio). The obtained cell suspension was spread onto clean, cooled, and wet slides. The specimens were code labeled and stained with 2% Giemsa solution (Merck).

The registration of metaphases (200 from each donor) included in the analysis and the criteria for cytogenetic damages were consistent with generally accepted recommendations^28^. The following parameters were taken into account: the proportion of aberrant metaphases (CAs), the frequency of chromatid-type aberrations (CTAs): сhromatid breaks and chromatid-type exchanges, and the frequency of chromosome-type aberrations (CSAs): chromosome breaks, dicentric and ring chromosomes, atypical monocentric chromosomes. Achromatic gaps were not included in the number of aberrations and were not taken into account.

### DNA extraction, 16S rRNA gene amplification and 16S rRNA gene sequencing

DNA was extracted from each sample using FastDNA Spin Kit For Soil (MP Biomedicals) based on the manufacturer’s recommendation. Thirty four 16S rRNA gene amplicon libraries were prepared by PCR amplification of an approximate 467 bp region within the hypervariable (V3-V4) region of the 16S rRNA gene in bacteria, from 50 ng of each of the extracted and purified DNA from sputum samples, respectively, according to the Illumina 16S metagenomic sequencing library protocol. PCR was initially performed with broad-spectrum 16S rRNA primers (forward primer: 5′-TCGTCGGCAGCGTCAGATGTGTATAAGAGACAGCCTACGGGNGGCWGCAG-3′, and reverse primer: 5′-GTCTCGTGGGCTCGGAGATGTGTATAAGAGACAGGACTACHVGGGTATCTAATCC-3′), using BioMaster Hi-Fi LR 2× 2× ReadyMix DNA polymerase (BiolabMix company, Novosibirsk, Russia). Cycle conditions were 94 °C (3 min 30 s), then 25 cycles of 94 °C (30 s), 55 °C (30 s), 68 °C (40 s), then a final extension of 68 °C (5 min). Libraries were purified using Agencourt AMPure XP beads (Beckman Coulter, Bray, USA) according to the Illumina 16 S metagenomic sequencing library protocol. Dual indices and Illumina sequencing adapters from the Illumina Nextera XT index kits v2 B and C (Illumina, San Diego, USA) were added to the target amplicons in a second PCR step using BioMaster Hi-Fi LR 2× 2× ReadyMix DNA polymerase (BiolabMix company, Novosibirsk, Russia). Cycle conditions were 94 °C (3 min 30 s), then 8 cycles of 94 °C (30 s), 55 °C (30 s), 68 °C (40 s), then a final extension of 68 °C (5 min). Libraries were again purified using Agencourt AMPure XP beads (Beckman Coulter, Bray, USA) according to the Illumina 16 S metagenomic sequencing library protocol. Sample PCR products were then pooled in equimolar amounts, purified using AMPure XP Beads (Beckman Coulter), and then quantified using a fluorometer (Quantus Fluorometer dsDNA (Promega, Madison, WI, USA). Molarity was then brought to 4 nM, the libraries were denatured, and then diluted to a final concentration of 8 pM with a 10% PhiX spike for sequencing on the Illumina MiSeq^29^.

### Taxonomy quantification using 16S rRNA gene sequences and statistical methods

The processing of the results was conducted with the help of the program QIIME2^30^. A quality check was carried out and a sequence library was generated. The sequences were combined into operational taxonomic units (OTUs) based on a 99% nucleotide similarity threshold using the Greengenes reference sequences library (versions 13–8) and SILVA (version 132), followed by the removal of singletones (OTUs containing only one sequence).

Calculation of indicators of alpha diversity and analysis of community similarity (beta diversity) was carried out according to the method UniFrac^31^. The total diversity of prokaryotic communities (alpha diversity) of sputum was estimated by the number of allocated OTU (analogue of species richness) and Shannon indices (H = Σp_i_ ln p_i_, p_i_ – part of i-sh species in community). When calculating sample diversity indices, 386 sequences were normalized (the minimum number of received sequences per sample). The variation in the structure of the bacterial community of different samples (beta diversity) was analyzed using UniFrac^31^ – a method common in microbial ecology that estimates the difference between communities based on the phylogenetic relationships of the presented taxa. In this study, we used a version of the unweighted UniFrac method that takes into account only the presence of taxa, but not their share in the community. The significance of differences between groups of samples was evaluated by the PERMANOVA method (Adonis).

In addition, to assess the significance of differences in the relative percentage of individual bacterial taxa in sputum, as well as the frequency of chromosomal aberrations in lymphocytes, the Mann-Whitney U test was used. To estimate the difference in the frequencies of occurrence, the Fisher exact test was used. Calculations were performed using the software package STATISTICA.10, Statsoft, USA.

## Results

### Characterization of sputum bacterial communities

**Figure 1:**
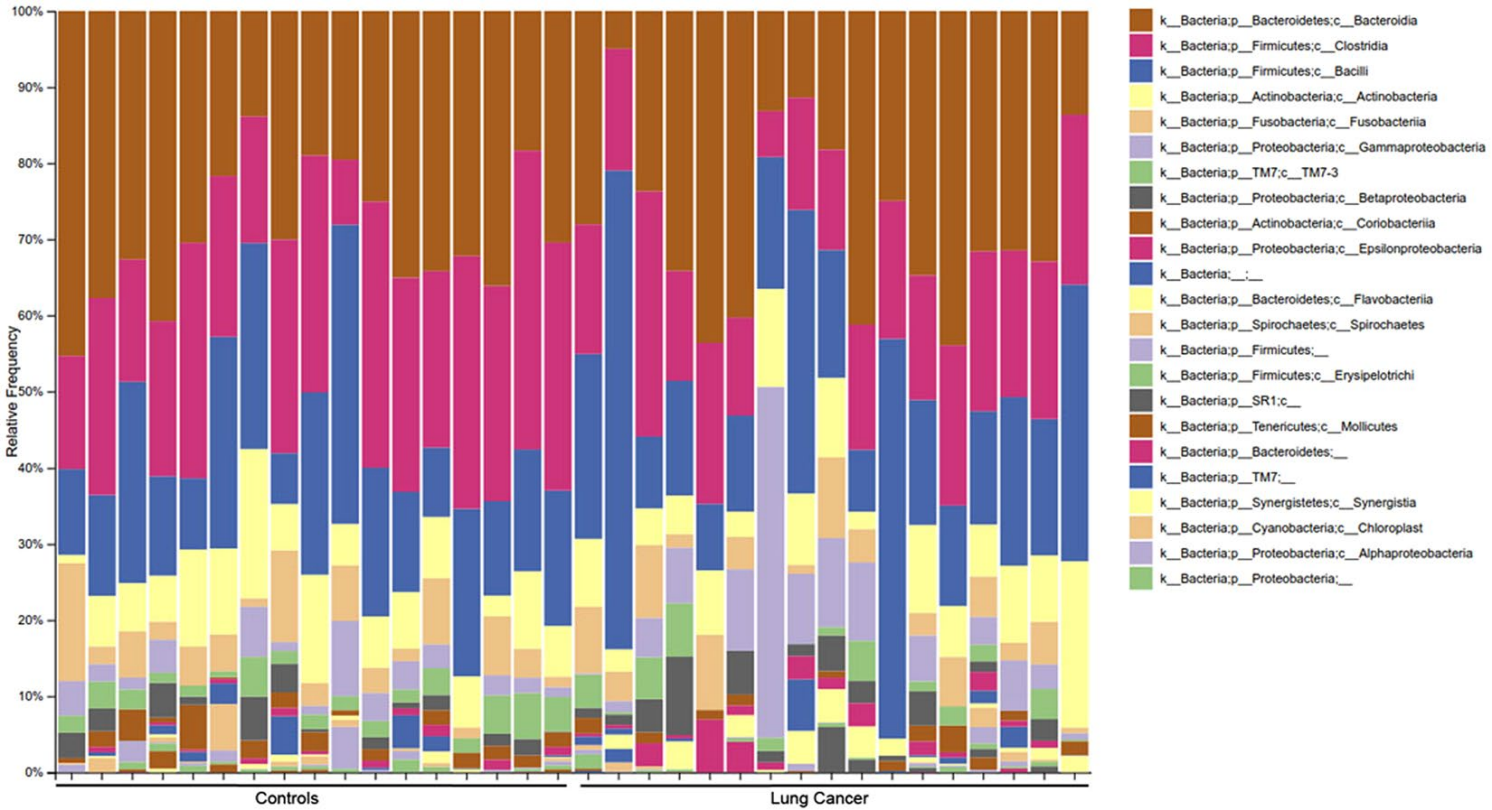
Taxonomic structure of microbiomes of upper respiratory tract among healthy and lung cancer patients.

In our sequencing approach (16S rRNA V3–V6) of LC and controls using sputum samples, we were able to identify a total of 11 phyla with relative frequencies above 0.1%. The prevailing phyla in our dataset were *Firmicutes*, *Bacteroidetes*, *Actinobacteria* and *Proteobacteria* (Fig. 1), as it could be expected from previous studies^15,18^.

Regarding alpha diversity, neither the number of allocated OTUs nor the Shannon indices, did not showed significant differences between LC and control. Overall, the bacterial communities were both fairly diverse as indicated by Shannon index at genus level (5.632 in LC vs 5.634 in controls). This suggests that any changes to the sputum microbiome as a result of a malignancy are not large-scale community shifts.

**Figure 2:**
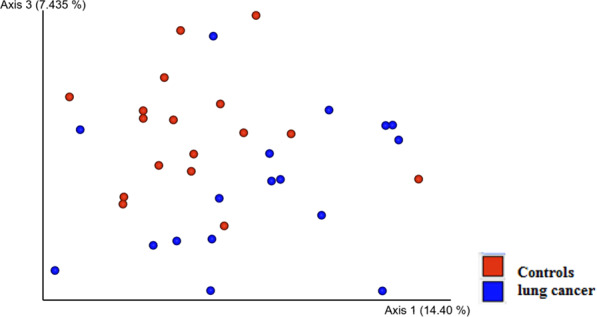
Phylogenetic similarity of prokaryotic sputum communities in LC patients and controls.

Differences in the structure of bacterial communities in sputum samples of lung cancer patients and controls are shown in Fig. 2. The PERMANOVA (Adonis) test using the difference matrix constructed by the unweighted UniFrac method showed a significant difference in the prokaryotic sputum communities of healthy people and LC patients (pseudo-F = 1.94; p = 0.005).

Sequencing statistics are summarised in Table 2 (for 67 genera) and in Table 3 (for 32 species), alongside corresponding U-rank Mann-Whitney p values. Among genera, *Streptococcus*, *Prevotella*, *Anaerosinus* and *Selenomonas* were the most common in the two pools. Seven genera (*Actinobacillus*, *Pediococcus*, *Abiotrophia*, *Ruminofilibacter*, *Elizabethkingia, Psychrobacter* and *Malus*) were found in lung cancer samples, but not in the control. Another eleven genera (*Alloscardovia*, *Lutibacter*, *Anaerorhabdus*, *Pyramidobacter*, *Barnesiella*, *Gardnerella*, *Corynebacterium*, *Scardovia*, *Olsenella*, *Eggerthella* and *Asholeplasma*) were found only in control samples, but not in lung cancer. Representatives of 13 genera were not found in the sputum of both groups: *Cardiobacterium*, *Defluviitalea*, *Finegoldia*, *Jonquetella*, *Mobiluncus*, *Olsenella*, *Rhodococcus*, *Rhizobium*, *Slackia*, *Sneathia*, *Sphingobacterium*, *Spirohaeta*, *Succinispira*. The data in Table 3 refers to the Greengenes reference sequences library (versions 13-8).

**Table 2:** Average percentage abundance of genera present in «core» microbiome.

Genus	Controls		LC		P Value
	Mean ± SD	Count	mean ± SD	Count	
**Found in LC and Controls samples**					
Streptococcus	15.68 ± 10.01	17/17	18.98 ± 17.79	17/17	>0.05
Prevotella (f. Prevotellfceae)	20.39 ± 6.58	17/17	17.77 ± 8.73	17/17	>0.05
Atopobium	2.2 ± 1.55	17/17	0.97 ± 0.9	12/17	**0.002**
Veillonella	13.79 ± 7.78	17/17	11.3 ± 4.05	17/17	>0.05
Anaerosinus	12.93 ± 6.99	16/17	11.07 ± 5.77	17/17	>0.05
Streptobacillus	3.51 ± 3.3	15/17	2.14 ± 2.27	13/17	>0.05
Selenomonas	5.54 ± 3.93	15/17	3.17 ± 2.58	16/17	>0.05
Prevotella (f. Paraprevotellacacea)	1.66 ± 1.9	12/17	1.81 ± 1.35	14/17	>0.05
Alloprevotella	3.6 ± 2.86	14/17	5.02 ± 3.09	17/17	>0.05
Lachnoanaerobaculum	0.61 ± 0.45	13/17	0.47 ± 0.8	8/17	>0.05
Megasphaera	2.67 ± 2.27	13/17	1.67 ± 1.65	12/17	>0.05
Stomatobaculum	1.13 ± 0.98	13/17	0.41 ± 0.49	8/17	**0.021**
Leptotrichia	2.63 ± 2.81	12/17	2.54 ± 2.84	14/17	>0.05
Solobacterium	0.45 ± 0.44	12/17	0.36 ± 0.46	11/17	>0.05
Gemella	1.8 ± 2.14	12/17	2.81 ± 2.18	15/17	>0.05
Fusobacterium	1.72 ± 1.8	11/17	1.69 ± 2.12	10/17	>0.05
Bacillus	1.67 ± 2.41	11/17	2.01 ± 2.0	13/17	>0.05
Campylobacter	0.53 ± 0.48	11/17	1.57 ± 1.74	15/17	>0.05
Actinomyces	1.91 ± 1.71	11/17	2.12 ± 2.13	12/17	>0.05
Treponema	0.55 ± 0.96	11/17	0.19 ± 0.49	3/17	**0.017**
Bulleidea	0.39 ± 0.43	11/17	0.33 ± 0.47	11/17	>0.05
Bacteroides	1.5 ± 1.65	10/17	3.23 ± 2.55	14/17	0.049
Macellibacteroides	1.33 ± 1.57	10/17	2.48 ± 2.34	11/17	>0.05
Clostridium (f. Lachnospiraceae)	0.62 ± 0.81	9/17	0.3 ± 0.52	6/17	>0.05
Granulicatella	1.55 ± 1.94	9/17	1.26 ± 1.62	8/17	>0.05
Porphyromonas	1.2 ± 1.78	9/17	0.14 ± 0.28	4/17	**0.03**
Capnocytophaga	0.26 ± 0.32	9/17	1.26 ± 1.53	10/17	>0.05
Oribacterium	0.67 ± 0.85	9/17	0.5 ± 0.61	11/17	>0.05
Vestibaculum	1.19 ± 1.95	8/17	0.53 ± 1.42	6/17	>0.05
Neisseria	0.9 ± 1.77	8/17	3.56 ± 5.16	11/17	>0.05
Dialister	0.35 ± 0.7	6/17	0.43 ± 0.55	10/17	>0.05
Filifactor	0.15 ± 0.24	6/17	0.06 ± 0.22	2/17	>0.05
Granulicatella	0.88 ± 1.31	6/17	1.19 ± 2.1	5/17	>0.05
Rothia	2.02 ± 2.02	5/17	1.63 ± 1.87	11/17	>0.05
Mycoplasma	0.36 ± 1.04	5/17	0.16 ± 0.38	5/17	>0.05
Moriella	0.34 ± 0.65	4/17	0.06 ± 0.16	2/17	>0.05
Clostridium (f. Clostridiaceae)	0.36 ± 0.96	3/17	0.09 ± 0.36	1/17	>0.05
Lactobacillus	0.15 ± 0.36	3/17	0.05 ± 0.15	2/17	>0.05
Bordetella	0.16 ± 0.43	3/17	0.14 ± 0.36	3/17	>0.05
Peptostreptococcus	0.25 ± 0.73	2/17	0.22 ± 0.7	2/17	>0.05
Bifidobacterium	0.18 ± 0.57	2/17	0.43 ± 1.43	4/17	>0.05
Bergeyella	0.06 ± 0.18	2/17	0.22 ± 0.27	8/17	**0.038**
Zhouea	0.02 ± 0.08	2/17	0.3 ± 0.85	3/17	>0.05
Catonella	0.04 ± 0.13	2/17	0.04 ± 0.12	2/17	>0.05
Haemophilus	0.05 ± 0.22	1/17	3.04 ± 9.78	8/17	**0.006**
Eggerthella	0.03 ± 0.11	1/17	0.08 ± 0.03	1/17	>0.05
Kocuria	0.04 ± 0.17	1/17	0.08 ± 0.19	3/17	>0.05
Shuttleworthia	0.02 ± 0.1	1/17	0.02 ± 0.07	2/17	>0.05
**Found only in LC samples**					
Actinobacillus	0	0/17	0.19 ± 0.47	4/17	**0.039**
Pediococcus	0	0/17	0.21 ± 0.75	2/17	>0.05
Abiotrophia	0	0/17	0.04 ± 0.17	1/17	>0.05
Ruminofilibacter	0	0/17	0.01 ± 0.03	1/17	>0.05
Elizabethkingia	0	0/17	0.02 ± 0.1	1/17	>0.05
Psychrobacter	0	0/17	0.01 ± 0.05	1/17	>0.05
Malus	0	0/17	0.003 ± 0.01	1/17	>0.05
**Found only in Controls samples**					
Alloscardovia	0.06 ± 0.17	2/17	0	0/17	>0.05
Lutibacter	0.04 ± 0.16	1/17	0	0/17	>0.05
Anaerorhabdus	0.03 ± 0.12	1/17	0	0/17	>0.05
Pyramidobacter	0.03 ± 0.11	1/17	0	0/17	>0.05
Barnesiella	0.02 ± 0.07	1/17	0	0/17	>0.05
Gardnerella	0.02 ± 0.07	1/17	0	0/17	>0.05
Corynebacterium	0.02 ± 0.07	1/17	0	0/17	>0.05
Scardovia	0.01 ± 0.05	1/17	0	0/17	>0.05
Olsenella	0.01 ± 0.04	1/17	0	0/17	>0.05
Eggerthella	0.01 ± 0.04	1/17	0	0/17	>0.05
g.Clostridium (f. Peptostreptococcacaea)	0.01 ± 0.05	1/17	0	0/17	>0.05
Acholeplasma	0.01 ± 0.05	1/17	0	0/17	>0.05

**Table 3:** Average percentage abundance of species present in «core» microbiome.

Species	Controls		LC		P Value
	Mean ± SD	Count	mean ± SD	Count	
Streptococcus agalactiae	16.5 ± 9.62	17/17	19.3 ± 17.56	17/17	<0.05
Atopobium rimae	2.27 ± 1.52	17/17	1.1 ± 0.91	13/17	**0.003**
Anaerosinus glycerini	13.11 ± 6.99	16/17	10.92 ± 5.51	17/17	>0.05
Selenomonas bovis	5.03 ± 4.13	15/17	2.75 ± 2.58	13/17	>0.05
Granulicatella balaenopterae	2.24 ± 1.59	15/17	2.56 ± 1.87	14/17	>0.05
Bacteroides nordii	1.71 ± 1.54	13/17	2.86 ± 2.22	14/17	>0.05
Lachnoanaerobaculum orale	0.69 ± 0.41	13/17	0.53 ± 0.79	10/17	>0.05
Megasphaera Micronuciformis	3.36 ± 2.46	13/17	2.32 ± 2.22	11/17	>0.05
Actinomyces hyovaginalis	2.04 ± 1.6	13/17	1.87 ± 1.49	12/17	>0.05
Prevotella Histicola	3.23 ± 3.68	12/17	1.35 ± 1.55	9/17	**0.04**
Prevotella pallens	2.13 ± 2.2	11/17	2.05 ± 2.21	11/17	>0.05
Bulleidia moorei	0.39 ± 0.43	11/17	0.33 ± 0.47	11/17	>0.05
Rothia terrae	1.95 ± 1.95	11/17	1.75 ± 2.06	10/17	>0.05
Prevotella sp. oral clone DO014	0.72 ± 0.68	11/17	0.27 ± 0.77	2/17	**0.009**
Prevotella Tannarae	1.0 ± 1.41	10/17	0.99 ± 1.15	11/17	>0.05
Macellibacteroides fermentans	1.49 ± 1.71	10/17	2.5 ± 2.29	13/17	>0.05
Treponema Amulovorum	0.27 ± 0.52	9/17	0.05 ± 0.17	2/17	**0.016**
Porphyromonas endodontalis	1.19 ± 1.78	9/17	0.23 ± 0.44	5/17	>0.05
Vestibaculum illigatum	1.22 ± 1.97	9/17	0.64 ± 1.45	7/17	>0.05
Prevotella nanceiensis	0.45 ± 0.7	8/17	0.86 ± 1.08	10/17	>0.05
Prevotella intermedia	0.81 ± 1.39	7/17	0.19 ± 0.39	4/17	>0.05
Prevotella nigrescens	0.76 ± 1.64	6/17	0.44 ± 0.55	8/17	>0.05
Clostridium bolteae	0.41 ± 0.66	6/17	0.31 ± 0.52	6/17	>0.05
Mycoplasma zalophi	0.31 ± 1.04	4/17	0.15 ± 0.28	4/17	>0.05
Moryella indoligenes	0.34 ± 0.65	4/17	0.06 ± 0.16	5/17	>0.05
Peptostreptococcus Anaerobius	0.26 ± 0.73	3/17	0.06 ± 0.26	1/17	>0.05
Clostridium Acidurici	0.34 ± 0.96	3/17	0.09 ± 0.36	1/17	>0.05
Lactobacillus Hamsteri	0.15 ± 0.36	3/17	0.05 ± 0.15	2/17	>0.05
Campylobacter rectus	0.07 ± 0.19	2/17	0.33 ± 0.49	7/17	>0.05
Bergeyella zoohelcum	0.03 ± 0.11	2/17	0.25 ± 0.25	10/17	**0.004**
Oribacterium Sinus	0.21 ± 0.66	2/17	0.13 ± 0.33	3/17	>0.05
Leptotrichia Trevisanii	0.01 ± 0.05	1/17	0.29 ± 0.78	3/17	>0.05

In the sputum of LC patients, compared to controls, there was a significant decrease in abundance by percentage of genus *Atopobium* (0.97 ± 0.9 vs 2.2 ± 1.55; p = 0.002); *Stomatobaculum* (0.41 ± 0.49 vs 1.13 ± 0.98; p = 0.021); *Treponema* (0.19 ± 0.49 vs 0.55 ± 0.96; p = 0.017). At the same time, the genus *Bergeyella* was significantly more represented in the microbiome of LC patients as compared to the controls (0.22 ± 0.27 vs 0.06 ± 0.18; p = 0.038). Representatives of the genus *Haemophilus* were observed only in 1 of 17 control donors (0.05 ± 0.22) and were present in sputum of 8 LC patients (3.04 ± 9.78; p = 0.006).

In LC, compared to controls, a decrease in the occurence of individual representatives of the same genera was observed at the species level: *Atopobium rimae* (1.1 ± 0.91 vs 2.27 ± 1.52; p = 0.003), *Treponema amulovorum* (0.27 ± 0.52 vs 0.05 ± 0.17; p = 0.016), as well as an increase in *Bergeyella zoohelcum* (0.25 ± 0.25 vs 0.03 ± 0.11; p = 0.004). Additionally, two different species from the genus *Prevotella* (*P.histicola* and *P. sp. oral clone DO014*) were significantly less represented in the sputum of patients than in the controls (Table 3).

Smoking status, as a factor that may have influenced the composition of the bacterial flora in LC patients and controls was studied separately. A significant difference in the occurrence of the species *Selenomonas bovis* in sputum was revealed in the samples of LC patients differing in smoking status (3.52 ± 2.55% in smokers and 0.91 ± 1.62% in non-smokers; p = 0.044); the genus *Bacteroides* (4.02 ± 2.51% in smokers and 1.35 ± 1.61% in non-smokers; p = 0.039); the genus *Zhouea* (0% in smokers and 1.03 ± 1.39% in non-smokers; p = 0.006); the genus *Selenomonas* (4.07 ± 2.42% in smokers and 1.03 ± 1.55% in non-smokers; p = 0.013); the genus *Peptostreptococcus* (0% in smokers and 0.73 ± 0.21% in non-smokers; p = 0.031).

Controls differing in smoking status revealed a significant difference in the occurrence of the species *Bulleidia moorei* in sputum (0.54 ± 0.45% in smokers and 0.12 ± 1.18% in non-smokers; p = 0.035); the genus *Granulicatella* (1.35 ± 1.43% in smokers and 0% in non-smokers; p = 0.04); genus *Neisseria* (0.39 ± 0.99% in smokers and 1.82 ± 2.54% in non-smokers; p = 0.026); genus *Bulleidea* (0.54 ± 0.46% in smokers and 0.13 ± 0.19% in non-smokers; p = 0.035).

Comparison of the species and generic microbiome compositions in the sputum of LC patients of the main pathomorphological forms (squamous, adenocarcinoma and large cell carcinoma) revealed significant differences in the content of the two bacterial genera *Veillonella* and *Leptotrichia*. In the sputum of patients diagnosed with large cell carcinoma, *Veillonella* representatives were recorded with a higher frequency than in patients with adenocarcinoma (15.35 ± 3.39% vs 7.68 ± 3.01%, respectively; p = 0.036). Representatives of the genus *Leptotrichia* were also recorded with a greater frequency in the sputum of patients with large-cell carcinoma as compared to adenocarcinoma (4.78 ± 2.81% vs 1.11 ± 1.13%, respectively; p = 0.036).

Comparison of the species and generic microbiome compositions in patients with different stages LC (I-II and III-IV) revealed a significant difference in only one species, *Porphyromonas endodontalis*, the content of which was significantly higher in the sputum of patients with stage I-II (0.53 ± 0.6%) compared with patients at the III-IV stage of RL (0.06 ± 0.21%; p = 0.021).

The presence of metastases to distant organs was associated with an increase in the content in the sputum of representatives of the genus *Capnocytophaga* (3.05 ± 1.58%) vs 0.71 ± 1.05% in LC patients without metastases (p = 0.016). In addition, *Atopobium rimae* showed a decrease in sputum in patients with metastases compared with patients without metastases (0.21 ± 0.43% vs 1.37 ± 0.84%, respectively; p = 0.017).

### Chromosomal aberrations in lymphocytes and sputum microbiome composition

Lung cancer patients had a significantly increased total frequency of CAs in comparison with controls (4.31 ± 1.86% *vs*. 2.27 ± 1.03%, p = 0.002). In LC patients, as compared to the control group, significant increases in the frequency of chromosome-type aberrations, such as сhromosome breaks (1.16 ± 0.94% *vs*. 0.38 ± 0.45%, p = 0.012) in comparison with the control group were detected (Table 4). No significant differences in chromatid-type aberrations were found between LC patients and controls. The Spearman correlation coefficient for the CAs, CTAs and CSAs frequencies with age (for the patients and the controls), were not significant (*p* > 0.05). No significant difference in the frequencies of CAs was detected between squamous cell lung cancer, adenocarcinoma, large cell lung carcinoma and TNM stage. Additionally, smoking status was not a factor significantly affecting the CAs, CTAs and CSAs frequencies for the two groups studied.

**Table 4:** Frequency of chromosome aberrations in lung cancer patients and controls.

Type of aberrations	Lung cancer patients (N = 17)		Controls (N = 17)		P-value
	Mean ±SD	Minimum–maximum	Mean ±SD	Minimum–maximum	
Aberrant metaphases	4.31 ± 1.86	1.5–7	2.27 ± 1.03	0.5–4.5	**0.002**
Chromatid breaks	2.63 ± 1.62	0–5.5	1.71 ± 1.06	0–3.5	0.13
Chromatid exchanges	0.16 ± 0.24	0–0.5	0.03 ± 0.12	0–0.5	0.067
Chromosome breaks	1.16 ± 0.94	0–3	0.38 ± 0.45	0–1.5	**0.012**
Chromosome exchanges	0.53 ± 1.01	0–4	0.29 ± 0.47	0–1.5	0.61

To analyze the possible effect of the taxonomic composition of the microbiome on the level of genetic instability in somatic cells of the host organism, based on the results of cytogenetic analysis, subgroups of patients and controls were formed that differed in the frequency of lymphocytes with CAs.

The subgroup with a low background level of CA (0–3.5%; the average value is 2.26 ± 0.86%) consisted of 7 patients and 16 control groups, and the subgroup with a high level of CA (more than 3.5%; the average value is −5.67 ± 1.15%) consisted of 9 patients and 1 control.

Comparison of the representation of Atopobium rimae type by percentage in the bacterial microbiome revealed a signficant decrease of Atopobium rimae in the sputum from the patients with lung cancer with a high level of CA as compared to the first subgroup (0.59 ± 0.63 versus 1.72 ± 0.9; p = 0.014).

In addition, we compared the content of bacterial genera and species in the combined subgroups of patients and controls with high and low levels of CA in lymphocytes.

**Figure 3:**
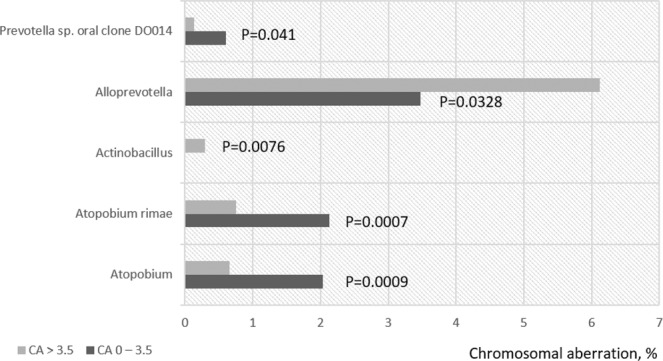
Representation of bacterial species and genera in the sputum of donors, differentiated by the level of chromosomal aberrations (CA, %) in blood lymphocytes.

In the composition of sputum from donors with high frequencies of CA frequencies, there is a significant decrease in representatives of the genus Atopobium and particularly of species of Atopobium rimae, as well as a decrease in representatives of Prevotella sp. oral clone DO014 (Fig. 3).

At the same time, an increase in the level of genetic instability in donor lymphocytes was associated with a significant increase in the content in the sputum of representatives of the genus Alloprevotella.

Representatives of the genus Actinobacillus were noted only in the sputum of donors with a high frequency of CA (0.29 ± 0.57%) and were not found in donors with a low level of aberrations in lymphocytes.

## Discussion

The contribution of the microbiota of the upper respiratory tract to the pathological transformation of the tissues of these organs is a recognized phenomenon in studies of such a widespread disease as LC.

The urgent problem remaining is the formation of adatabase that is as complete as possible on the microbiome of the respiratory organs of people from different geographical regions of the planet, the establishment of strong cause-effect/associative relationships, and the identification of the molecular mechanisms of LC pathogenesis. This will open up prospects for early diagnosis and, ultimately, open up new approaches in the treatment of LC.

This pilot study, for the first time, presents the results of an investigation of the microbiome of the respiratory tract in a small group of patients with lung cancer of various histological types and degree, residing in Kuzbass, an industrial region of Russia.

Through the use of 16S metagenomic sequencing, we determined differences in the composition of the microbiome up to the species level in 17 men with a confirmed diagnosis of lung cancer (LC) compared with 17 age-matched controls.

In our study, we found 12 types, 119 genera, 118 OTU and analyzed 187361 sequences in total.

The taxonomic structure of the upper respiratory tract microbiome at the type level is shown in Fig. 1, from which it follows that the microbiome is represented mainly by representatives of the Bacteroidetes, Firmicutes, Actinobacteria types.

At the species level, differences in the composition of the microbiome in sputum in patients with LC and in control donors (Table 4) are characterized by a statistically significant decrease in the prevalence of four types of bacteria, namely: *Prevotella histicola*, *Prevotella sp. oral clone DO014*, *Atopobium rimae*, *Treponema amylovorum* and increase in *Bergeyella sp. AF14* (*Flavibacteriaceae*) species in LC patients.

*Prevotella histicola* and *Prevotella sp. oral clone DO014* belong to type *Bacteroidetes*, whose family members were recently associated with a worse prognosis for patients with LC^32^. Although overrepresented in LC patients on a type level, an individual species still could be underepresented in patients with LC, such as Prevotella sp. oral clone DO014 in our case, by analogy with *Filifactor* species. *Filifactor* bacteria were recently discovered mainly in healthy study participants and was even noted as a good control that allowed to distinguish between healthy and LC patients^11^, while the type where *Filifactor* belongs is represented more frequently in patients with lung cancer^13^. We also detected *Filifactor* mainly in healthy people.

Our data are consistent with previously published data. Representatives of the genera Treponema and *Filifactor* were found as ideal biomarkers in healthy participants in LC study^11^.

Representatives of the genus *Atopobium* are usually found in the oral cavity. *Atopobium rimae* species are associated with chronic periodontitis, bacteria of this species can cause bacteremia, are strictly anaerobic and Gram-positive bacteria, short and elliptical in shape, with a low content of G + C nucleotides^33^. The association of this species with LC has not been noted previously.

In our study, representatives of the genus *Stomatobaculum* are less represented in the sputum of patients with LC. This species has only recently been described as part of the microbiota of the human oral cavity and is positively associated with chalicytosis^34–36^, and with increased fasting glucose level (which is a sign of an increase in blood glucose after 2 years and the development of insulin insensitivity) It is also associated with a slowdown in bone restoration during implantation^37^. *Stomatobactulum* is strictly anaerobic, Gram-positive, non-spore forming, contains inclusions of iron and sulfur. For this species as well, association with LC was not previously noted.

Sequencing of bacterial 16S rRNA genes from the sputum of the lung cancer patients in our study has shown that the genera *Atopobium* and *Stomatobaculum* are significantly less abundant in samples from lung cancer patients as compared to the controls. The association of these two genera with the development of LC was not previously noticed. However, it was previously reported that the family *Lachnospiraceae*, to which *Stomatobaculum* belongs showed a positive relationship with LC^32^.

The representation of bacterial species of *Atopobium rimae* in the sputum of patients with LC decreases with progression of metastases according to our study, but this requires confirmation in a larger sample collection.

Our 16S rRNA sequencing data indicate an increase in the content of bacteria of the species *Bergeyella sp. AF14* (*Flavibacteriaceae*) in the sputum of LC patients with. Previously, this type of bacteria was not associated with LC. Published data link this species of bacteria to animal respiratory disease^38,39^. Only recently have these bacteria have been identified as the cause of infectious endocarditis in humans^40^.

Interestingly, *Bergeyella* is represented to a lesser extent in patients with chalicytosis^34^. To firmly establish a more reliable association of this bacterium with the diagnosis of LC, a srtudy with more participants will be required.

Regarding the detection of such widespread bacteria in the respiratory tract as *Haemophilus inflluenzae* in our samples, we were able to detect it by analyzing the bacterial DNA 16S rRNA gene sequences using the SILVA database, which is based on 16S/18S *Archaea* analysis. We see that *Haemophilus inflluenzae* is present in 17 patients with LC and only in 1 subject in the control (p = 0.016). This contradicts previously published data that *Haemophilus inflluenzae* is mainly detected in healthy individuals^15^. It is known that most strains of *H. influenzae* are opportunistic pathogens that coexist with the host without causing disease, and only concomitant factors such as viral infections, decreased immunity, or chronically inflamed tissues such in allergies, predispose for pathogenic infections with *H. influenzae*. Thus, the reproduction of this pathogen and, therefore, higher chances of its detection should be expected precisely in the case of LC, as our data show. In any case, this discrepancy in the data deserves further study, with the involvement of more participants in the study of microbiota of the upper respiratory tract.

The frequencies of bacterial genera detected in our study indicates a decrease in the diversity of the microbiome of LC patients as compared to controls (Table 3). Seven representatives of unique bacterial genera were found in patients with LC compared to 14 in the controls.

Our data are consistent with previously published data showing that the lung microbiota in LC patients has a less complex composition. LC patients are distinguished by a decrease in the alpha diversity of the lung microbiota^41^.

Comparison of the microbiome composition in the sputum of patients with lung cancer of the main histological types (squamous cell carcinoma, adenocarcinoma and large-cell carcinoma) at the species and genera level did not reveal significant differences, except for the occurrence of bacteria of the genus *Bergeyella*. In the sputum of patients diagnosed with adenocarcinoma, representatives of *Bergeyella* were recorded at a higher frequency than in patients with squamous cell carcinoma (0.4 ± 0.24% and 0.04 ± 0.1%, respectively; p = 0.045).

Comparison of the microbiome in the sputum of patients with lung cancer of different stages (I-II and III-IV) at the species and genera level did not reveal significant differences.

Comparison of the microbiome in the sputum of patients with lung cancer with metastases in distant organs and without them in distant organs at the species and genera level did not revealatopobium significant differences, with the exception of the genus *Atopobium*, whose representatives were much less frequently detected in patients with metastases (0.21 ± 0.43% and 1, 37 ± 0.084%, respectively; p = 0.017), in particular, species of *Atopobium rimae* (0.21 ± 0.43% and 1.21 ± 0.089%, respectively; p = 0.034).

Interestingly, there was a different and non overlapping representation of bacterial species in the sputum microbiome of smokers as compared to non smokers in both LC patients and healthy participants in the study.

We examined the chromosome aberrations in the blood lymphocytes of all participants in the study and found that there is a significant correlation between the level of bacteria of a certain species in the sputum and chromosomal aberrations (CA).

Representatives of the genus *Alloprevotella* were most frequently associated with chromosomal aberrations in all participants in our study (Fig. 3). Bacteria of this genus have recently been identified as reliably associated with the metastatic stage of melanoma^42^.

Interestingly, representatives of the genus Actinobacillus were found only in the group of participants with high levels of CA. Representatives of the genus *Actinobacillus* (*A.ureae* and *A. hominis)* were previously found in the respiratory tract of healthy people and can cause bronchopneumonia and meningitis^43^.

Previous publications reported an increase in chromosome damage in the lymphocytes of primary LC patients^44–46^. The results obtained in our study confirm this (Table 4).

This increase in the level of cytogenetic lesions cannot be explained by the consequences of radiation or chemical therapy, since biological material from patients was collected before any treatment procedures. Instead, the cytogenetic instability of somatic cells in untreated patients with LC is a sign that reflects the influence of endogenous genotoxic factors on the human genome. In particular, one such factor can be oxidative stress, which is an indispensable attribute of the tumor process. In addition, the possible clastogenic effects of the bacterial environment in the lung tissue cannot be ruled out as causative agent. The influence of lung microbiota on lung carcinogenesis, immunity and immunotherapy is summarized in a recent review, the major points of which agree with our results as follows: the microbiota of the healthy lung is different from neoplastically transformed lung; bacterial products might promote host oncogene activation; the lung immune system is under the influence of the microbiota^47^.

It would be interesting to conduct a screening of the microbiome of the respiratory tract on a larger scale and for a longer period of time, for example, over three years, in order to identify the types of bacteria detected by us among healthy people from the same age group and region, in order to compare the data with the subsequent diagnosis of lung cancer and other diseases of the respiratory tract.

We plan to confirm the representation of different bacterial species detected by us in the sputum of patients with LC and healthy subjects by other methods, for example, by the method of specific quantitative PCR.

## Conclusion

The method of mass parallel sequencing of 16S ribosomal genes was used to determine the taxonomic composition of the microbiome in the sputum of LC patients and healthy donors (for the first time in the Russian population). Bacterial taxonomic groups have been identified where the microbiome composition differs significantly in patients as compared to controls. The discrepancy between our data and the results of previous studies of the LC microbiome probably reflects the specific epidemiological circumstances of the environment in the population we studied, a region with intensive coal mining and processing. The results obtained for the taxonomic composition of the microbiome rely on experimental data that will be further confirmed using a larger number of patients and control groups. The sputum microbiome, although it does not reflect the specific location of the respiratory tract, can potentially serve as an important non-invasive biomarker in LC. Our results show a correlation between chromosomal aberration of host genomes with bacterial representation in pharyngeal microbiome.

Thus, a comparison of the bacterial composition in the sputum of donors with cytogenetic damages in theirs lymphocytes, warrants further investigations on the potential role of microorganisms in the process of mutagenesis in somatic cells of the host body.

Knowledge of the specific composition of the microbiota in the respiratory tract of a patient will make it possible to predict the effectiveness of chemoradiotherapy, gene therapy, immunotherapy and other treatment methods, and may also contribute to the development of innovative strategies for early prevention and personalized treatment of lung cancer.
